# Supplementary material for: Content-rich biological network constructed by mining PubMed abstracts
Source: BMC Bioinformatics. 2004 Oct 8;5:147. doi: 10.1186/1471-2105-5-147 (PMC528731; doi:10.1186/1471-2105-5-147)
Supplement: Additional File 2 — The original results of the above study (non-essential files are deleted to keep the file size under the limit set by BMC bioinformatics). [file 1471-2105-5-147-S2.bz2 › chilibotAdditionalFile2/dip50/292ID9485448E1123/html/TRPA_TRPB.html]

 


 **TRPA** and **TRPB** 
  
Found 55 abstracts in PubMed, retrieved 50.  
 

 What does Google say? 
 PDF only 
| .edu only 

---

**Interactive relationship** (e.g. stimulation, inhibition, etc)

**Neutral relationship**- Previous studies demonstrated that the  **trpA**  Shine Dalgarno sequence within  **trpB**  and or the location of the  **trpB**  stop codon influenced  **trpA**  expression.  Ref: 2685759 Nucleic Acids Res, 1989
- The proximity of the  **trpB**  stop codon to the  **trpA**  start codon therefore markedly influences  **trpA**  expression.  Ref: 2685759 Nucleic Acids Res, 1989
- When a 4 bp overlapping leader ORF was placed in front of the successive  **trpB**  and  **trpA**  genes, the  **trpA**  gene was poorly expressed whereas the  **trpB**  gene was overexpressed.  Ref: 12097161 J Biochem Tokyo , 2002
- The third ORF ORF3 consisted of the first 77 nucleotides of the gene encoding the alpha subunit of tryptophan synthase  **trpA** , and overlapped the 3 end of  **trpB**  by 14 nucleotides.  Ref: 8581158 Microbiology, 1996
- Efficient translation of the  **trpA**  coding region is subject to translational coupling, i.e., maximal  **trpA**  expression is dependent on prior translation of the  **trpB**  coding region.  Ref: 2685759 Nucleic Acids Res, 1989
- Considering the primitive characteristics of C. caldarium,  **trpB**  must have been lost from the plastid genome before  **trpA** .  Ref: 8082179 Curr Genet, 1994
- Expression of  **trpB**  and  **trpA**  of the Escherichia coli tryptophan operon is shown to be translationally coupled, i.e., efficient translation of the  **trpA**  coding region is dependent on prior translation of the  **trpB**  coding region and termination of translation at the  **trpB**  stop codon.  Ref: 6377236 Nucleic Acids Res, 1984
- A comparison of the deduced amino acid sequence of the trp 3 polypeptide with its homologs in Saccharomyces cerevisiae encoded by the TRP5 gene and Escherichia coli encoded by the  **trpA**  and  **trpB**  genes shows that the A and B domains amino acid segments homologous to the  **trpA**  and  **trpB**  polypeptides, respectively of the N. crassa and yeast polypeptides are in the same order NH2 A B COOH.  Ref: 2521855 J Biol Chem, 1989
- The amino acid sequences deduced from the nucleotide sequences of Thermus  **trpB**  and  **trpA**  were found to have identities of 54.8 and 28.7%, respectively, with those of E. coli  **trpB**  and  **trpA**  genes.  Ref: 2188962 J Bacteriol, 1990
- However, neither the  **trpA**  gene nor the  **trpB**  gene of L. casei can complement mutations in the E. coli  **trpA**  gene and the  **trpB**  gene, respectively, suggesting that the protein products of the L. casei and E. coli  **trpA**  and  **trpB**  genes, respectively, cannot form heterodimers of tryptophan synthetase with activity.  Ref: 2113923 J Biochem Tokyo , 1990
- These findings suggest that removal of helix 0 of  **TrpA**  decreases  **TrpA**   **TrpB**  association as well as the activity of the  **TrpA**  active site.  Ref: 8662916 J Biol Chem, 1996

**Non-interactive relationship** (e.g. studied together, co-existance, homology, etc.)

- From 500 members of the gene library, clones were identified which complemented trpE, glnA and Thr mutations in E. coli but none which complemented trpD, trpC,  **trpB** ,  **trpA** , proA or Leu mutations.  Ref: 2887629 J Gen Microbiol, 1986
- The physical maps of these plasmids establish the gene order promoter  **trpB**   **trpA** .  Ref: 6260735 J Bacteriol, 1981
- The mutants belong to two different complementation groups,  **trpA**  and  **trpB** , which complement each other in heterokaryons.  Ref: 2615762 Mol Gen Genet, 1989
- Five trp genes, trpD, trpC, trpF,  **trpB** , and  **trpA** , of Lactobacillus casei were cloned by transformation of tryptophan auxotrophic mutants of the respective trp genes in Escherichia coli.  Ref: 2113923 J Biochem Tokyo , 1990
- Poor activity in each reaction was partly due to reduced association of  **TrpA**  with  **TrpB** .  Ref: 8662916 J Biol Chem, 1996
- The  **trpB**  and  **trpA**  coding regions of the polycistronic trp mRNA of Escherichia coli are separated by overlapping translation stop and start codons.  Ref: 2685759 Nucleic Acids Res, 1989
- Following the nomenclature established for E. coli and Serratia marcescens, the B. lactofermentum trp genes were designated trpL, trpE, trpG, trpD, trpC including the trpF domain,  **trpB** , and  **trpA** .  Ref: 2823076 Mol Gen Genet, 1987
- The alpha and beta reaction activities of independently purified Tk  **TrpA**  and Tk  **TrpB**  were 10 fold lower than the respective activities detected from the Tk TS complex, indicating that during heat treatment, each subunit was necessary for the other to obtain a proper conformation for high enzyme activity.  Ref: 11029579 Eur J Biochem, 2000
- By complementation tests using subcloning of the 9.6 kb NcoI fragments and various E. coli tryptophan auxotrophs, this fragment was found to contain a gene cluster composed of trpE, trpD, trpC,  **trpB**  and  **trpA**  in this order.  Ref: 8956524 Proc Natl Sci Counc Repub China B, 1996
- Complementation of the C. crescentus trp mutants indicated that the tet promoter was not necessary for expression in C. crescentus and suggested that at least two native promoters were present for expression of the trpF,  **trpB** , and  **trpA**  genes.  Ref: 6090420 J Bacteriol, 1984
- The trpE, trpD, trpC, trpF,  **trpB**  and  **trpA**  mutants were identified.  Ref: 4043728 Genetika, 1985
- In contrast to  **trpA** ,  **trpB**  the gene for the beta subunit of tryptophan synthase was encoded in the cell nucleus.  Ref: 8082179 Curr Genet, 1994
- Transcription of the cloned genes took place from their own promoters, in the following order  **trpB**  > **trpA** .  Ref: 1294446 Genetika, 1992
- To investigate possible explanations for the apparent transposition that occurred in the evolution of the fungal gene we have made two fusions of  **trpB**  and  **trpA**  of Escherichia coli in their natural orientation.  Ref: 2957370 J Biol Chem, 1987
- The trpF,  **trpB** , and  **trpA**  genes were on one cosmid, while the trpD and trpC genes were on two separate cosmids.  Ref: 2838460 J Bacteriol, 1988
- Gel filtration chromatography revealed that Tk  **TrpA**  was a monomer alpha, Tk  **TrpB**  was a dimer beta2, and Tk TS complex was a tetramer alpha2 beta2.  Ref: 11029579 Eur J Biochem, 2000
- This lack of regulation of the trpE, trpF,  **trpB** , and  **trpA**  genes is consistent with the idea that oligotrophic bacteria, such as C. crescentus, do not utilize regulatory mechanisms that greatly alter the biosynthetic capabilities in exponentially growing cells.  Ref: 2448292 J Bacteriol, 1988
- Precursor feeding experiments, accumulation studies, and complementation experiments resulted in the identification of six genes corresponding to  **trpA** ,  **trpB** , trpC, trpD, trpE, and trpF.  Ref: 6090420 J Bacteriol, 1984
- The polymerase chain reaction PCR was applied to clone the luxA and luxB genes from Vibrio harveyi, and the trp poL promoter operator leader region and the  **trpB**  and  **trpA**  genes from Escherichia coli.  Ref: 2034229 Mol Gen Genet, 1991
- In this study it is shown that, under conditions of intense selection, a strain carrying missense mutations in both  **trpA**  and  **trpB**  reverts to Trp 10 8 times more frequently than would be expected if the two mutations were the result of independent events.  Ref: 2062865 Proc Natl Acad Sci U S A, 1991
- 5 MTR regulatory mutants are characterized by depression of trpE, trpD and trpC genes, which causes the production of intermediates of tryptophan biosynthesis and results in  **trpA**  and  **trpB**  genes induction as well as in two fold activation of N 5 phosphoribosyl anthranilateisomerase trpF gene product.  Ref: 3957024 Genetika, 1986
- Codon usage in these Pseudomonas genes shows a marked preference for codons ending in G or C, thereby resembling that of  **trpB** ,  **trpA** , and several other chromosomal loci from this species and others with a high G C content in their DNA.  Ref: 3127654 Mol Biol Evol, 1986
- Subcloning and complementation of Escherichia coli trp auxotrophs revealed that as in Brevibacterium lactofermentum, the C. glutamicum trp genes are clustered in an operon in the order trpE, trpD, trpC,  **trpB** ,  **trpA** .  Ref: 7683184 Appl Environ Microbiol, 1993
- Nucleotide sequences of the trpI,  **trpB** , and  **trpA**  genes of Pseudomonas syringae positive control unique to fluorescent pseudomonads.  Ref: 8423001 Gene, 1993
- To overexpress broad kinds of GC rich genes in Escherichia coli, we examined how the structures of leader open reading frames leader ORFs affect the expression of GC rich genes, such as polA,  **trpA** , and  **trpB** , from Thermus thermophilus.  Ref: 12097161 J Biochem Tokyo , 2002
- Both pZAZ5 and pZAZ167 display Pseudomonas like regulation of the  **trpA**  and  **trpB**  genes.  Ref: 6260735 J Bacteriol, 1981
- B domains have 75% identity, and somewhat weaker homology to the E. coli  **trpA**  and  **trpB**  polypeptides A domains have 31% identity.  Ref: 2521855 J Biol Chem, 1989
- Differences in patterns of codon usage distinguish the trpI genes of P. syringae and P. putida from P. aeruginosa trpI and from the  **trpB**  and  **trpA**  genes of all three species.  Ref: 8423001 Gene, 1993
- Translational coupling of the  **trpB**  and  **trpA**  genes in the Escherichia coli tryptophan operon.  Ref: 6319355 J Bacteriol, 1984
- The  **trpA**  and  **trpB**  genes are inducible by indol 3 glycerophosphate.  Ref: 4065557 Genetika, 1985
- The arrangement of genes in the trpCBA cluster is unusual in that trpC is separated by a small open reading frame, trpX, from the potentially translationally coupled  **trpB**  and  **trpA**  genes.  Ref: 10361288 Mol Microbiol, 1999
- Promoters are fused to the 3 end of  **trpA**  rather than the HindIII site at the 5 end of  **trpB**  used in other vectors containing the W205 deletion.  Ref: 2999851 Plasmid, 1985
- To examine the dependence of  **trpA**  expression on the ribosome binding site sequence in the distal segment of  **trpB** , deletions were produced that replaced this  **trpB**  sequence.  Ref: 6377236 Nucleic Acids Res, 1984
- Restoration of a translational stop start overlap reinstates translational coupling in a mutant  **trpB**   **trpA**  gene pair of the Escherichia coli tryptophan operon.  Ref: 2685759 Nucleic Acids Res, 1989
- The structures of the C. crescentus usg, trpF,  **trpB** , and  **trpA**  genes were further analyzed in terms of codon usage, G C content, and genetic signals and were related to genetic signals previously identified in C. crescentus and other bacteria.  Ref: 2828322 J Bacteriol, 1988
- Unusual  **trpA**  and  **trpB**  auxotrophs with TrpAB phenotype were described.  Ref: 4043728 Genetika, 1985
- We conclude from these results that if the  **trpB**  gene is not translated, efficient translation of the  **trpA**  lac Z mRNA does not occur and, thus, that these genes are translationally coupled.  Ref: 6319355 J Bacteriol, 1984
- The trpF,  **trpB** , and  **trpA**  genes appeared to be linked, but they were not closely associated with trpD or trpC genes.  Ref: 2838460 J Bacteriol, 1988
- A 904 bp probe from Pseudomonas aeruginosa was used to identify the  **trpB** ,  **trpA**  and trpI genes of Pseudomonas syringae.  Ref: 8423001 Gene, 1993
- Complementation analysis in Escherichia coli strains, with defined mutations in tryptophan biosynthesis, and in vitro enzyme activity data, with cloned  **TrpB**  and  **TrpA**  proteins, indicate these mutations result in a  **TrpA**  protein that is unable to utilize indole glycerol 3 phosphate as substrate.  Ref: 12011099 J Biol Chem, 2002
- the former expresses  **trpB**  but not  **trpA** , and the latter has lost both activities.  Ref: 6260735 J Bacteriol, 1981
- Unexpectedly,  **trpA**  was not found downstream of  **trpB** .  Ref: 10511537 Genetics, 1999
- The trp poL was used successfully to control the expression of lac alpha, luxAB,  **trpB**  and  **trpA** .  Ref: 2034229 Mol Gen Genet, 1991
- One of the most puzzling examples of selection induced mutations involved the simultaneous reversions of two mutations, one in  **trpA**  and the other in  **trpB** , at rates that were several orders of magnitude greater than would have been predicted if the two mutations had occurred as independent events B.  Ref: 8407817 J Bacteriol, 1993
- When  **trpB**  translation proceeded in the wild type reading frame and terminated at the normal  **trpB**  stop codon,  **trpA**  polypeptide levels were elevated over the levels observed when translation stopped before or after the natural  **trpB**  stop codon.  Ref: 2685759 Nucleic Acids Res, 1989
- This high conservation supports the hypothesis of a common ancestor for the  **trpA**  and  **trpB**  genes of archaebacteria, eubacteria and eucaryotes.  Ref: 3146017 Mol Gen Genet, 1988
- One of these clones contained the genetic information needed to complement  **trpA**  and  **trpB**  mutations in Escherichia coli.  Ref: 2110092 FEMS Microbiol Lett, 1990
- The trp genes were classified into three linkage groups and they all were closely linked on a short chromosomal region probably in the order  **trpA** ,  **trpB** , trpF trpC, trpD trpE.  Ref: 8586278 FEMS Microbiol Lett, 1995
- The DNA sequences of the Caulobacter crescentus trpF,  **trpB** , and  **trpA**  genes were determined, along with 500 base pairs bp of 5 flanking sequence and 320 bp of 3 flanking sequence.  Ref: 2828322 J Bacteriol, 1988
- Fusion of  **trpB**  and  **trpA**  of Escherichia coli yields a partially active tryptophan synthetase polypeptide.  Ref: 2957370 J Biol Chem, 1987
- Overexpression in Escherichia coli of the AT rich  **trpA**  and  **trpB**  genes from the hyperthermophilic archaeon Pyrococcus furiosus.  Ref: 12435500 FEMS Microbiol Lett, 2002
- S1 nuclease mapping of in vivo transcripts indicated that usg, trpF,  **trpB** , and  **trpA**  are arranged into a single operon, with the transcription initiation site located 30 bp upstream from the start of usg.  Ref: 2828322 J Bacteriol, 1988
- The  **trpA**  and  **trpB**  genes encoding the tryptophan synthase subunits were cloned from the hyperthermophilic archaeon Pyrococcus furiosus.  Ref: 12435500 FEMS Microbiol Lett, 2002
- This plasmid expresses  **trpA**  and  **trpB**  constitutively.  Ref: 6260735 J Bacteriol, 1981
- Missing enzyme activities allowed assignment of all mutant to the trpE, trpD,  **trpB** , or  **trpA**  gene, confirming earlier results with the same mutants Johnston et al.  Ref: 7061383 J Bacteriol, 1982
- To explore the functional role of this region, we analyzed the effects of 19 single and double mutations in  **TrpB**  on the tryptophan synthase TSase and serine deaminase SDase activities of the TrpB2 dimer, either in the presence or in the absence of the  **TrpA**  subunit.  Ref: 11209753 Biol Chem, 2000
- Structural studies have identified residues 277 283 of  **TrpB**  as a potentially important region for the allosteric communication between the  **TrpA**  and  **TrpB**  subunits and for the transport of indole between their active sites through a hydrophobic tunnel.  Ref: 11209753 Biol Chem, 2000
- The chromosomal order of all bacterial genes that specify tryptophan synthetase beta and alpha chains, respectively, is  **trpB**   **trpA** .  Ref: 2957370 J Biol Chem, 1987
- The other complements  **trpA** ,  **trpB**  and trpC mutations in E. coli.  Ref: 2110092 FEMS Microbiol Lett, 1990
- Three pairs of genes trpG trpD, trpC trpF, and  **trpB**   **trpA**  overlap, and there is intercistronic spacing of 12 6, and 585 bp between the trpE trpG, trpD trpC, and trpF  **trpB**  gene pairs, respectively.  Ref: 1400208 J Bacteriol, 1992
- Plasmid pEP3923 7.4 kb comprises intact  **trpB**  and  **trpA**  genes and single BglII, HindIII, EcoRI, SalI and XhoI sites.  Ref: 6247248 Gene, 1980
- Hybrid tryptophan synthetase alpha and beta polypeptides were produced by genetic recombination between the  **trpB**   **trpA**  regions of Escherichia coli and Salmonella typhimurium contained on compatible, multicopy plasmids.  Ref: 7017727 Proc Natl Acad Sci U S A, 1981
- The tryptophan synthases from Escherichia coli and Salmonella typhimurium are tetrameric enzymes, with an elongated  **TrpA** . **TrpB** . **TrpB** . **TrpA**  structure.  Ref: 11209753 Biol Chem, 2000
- The Pseudomonas aeruginosa tryptophan synthase genes,  **trpA**  and  **trpB** , which are induced by their substrate indoleglycerol phosphate, were cloned along with their controlling region into the BamHI site of pBR322 to produce the 10.7 megadalton plasmid pZAZ5.  Ref: 6260735 J Bacteriol, 1981
- Subcloning, complementation of trp strains, nucleotide sequencing of 5.1 kb and 1.95 kb of DNA and subsequent homology comparisons identified the trpC,  **trpB**  and  **trpA**  genes and trpD gene respectively.  Ref: 10361288 Mol Microbiol, 1999
- The enzyme, which is produced by a multicopy plasmid encoding the  **trpA**  and  **trpB**  genes of S. typhimurium expressed in Escherichia coli, is crystallized from crude extracts by the addition of 6% poly ethylene glycol 8000 and 5 mM spermine.  Ref: 2495283 J Biol Chem, 1989
- We investigated whether there is translational coupling between the tryptophan operon  **trpB**  and  **trpA**  genes in Escherichia coli.  Ref: 6319355 J Bacteriol, 1984
- The presence of indole, the substrate for the terminal trp pathway enzyme tryptophan synthase, reversed this auxotrophy thereby providing evidence for the existence of an internal promoter independently expressing the tryptophan synthase genes  **trpB**  and  **trpA** .  Ref: 8093062 Biochem Biophys Res Commun, 1994
- Thus,  **trpB**  and  **trpA**  are regulated positively in three species of fluorescent pseudomonads, P. aeruginosa, P. putida, and P. syringae, but in no other eubacteria so far investigated Crawford, Annu.  Ref: 8423001 Gene, 1993
- Surprisingly, concomitant increases in mRNA levels were not detected from the trpF,  **trpB** , or  **trpA**  coding regions downstream in the operon.  Ref: 2448292 J Bacteriol, 1988
